# Retraction: Population genetics of 24 Y‐STR loci in Chinese Han population from Jilin Province, Northeast China

**DOI:** 10.1002/mgg3.2367

**Published:** 2024-02-12

## Abstract

Xu X‐m., Zheng J‐L., Lou Y., Wei X‐h., Wang B‐j.,Yao J. (2019). Population genetics of 24 Y‐STR loci in Chinese Han population from Jilin Province, Northeast China. *Molecular Genetics & Genomic Medicine*, 7(11), e984. https://doi.org/10.1002/mgg3.984

The above article, published online on 30 September 2019 in Wiley Online Library (wileyonlinelibrary.com), has been retracted by agreement between the journal Editor in Chief, Suzanne Hart, and Wiley Periodicals LLC. Following publication of this article, concerns were raised by a third party regarding consent and ethical approval for the research undertaken and reported in the article. The publisher and representatives of the journal’s editorial board undertook a review of the consent documentation provided, against the research performed and reported in the article. The review uncovered inconsistencies between the consent documentation and the research reported; the documentation was not sufficiently detailed to resolve the concerns raised. As a result, the parties have made the decision to retract the article. In addition, consent documentation did not give approval for data associated with this article to be shared publicly. The associated data for this article has been withdrawn from the publication record following a concern that the data could be used to identify those who have participated in the research. Jun Yao responded on behalf of all the authors, who disagree with the retraction.

Xu X‐m., Zheng J‐L., Lou Y., Wei X‐h., Wang B‐j.,Yao J. (2019). Population genetics of 24 Y‐STR loci in Chinese Han population from Jilin Province, Northeast China. *Molecular Genetics & Genomic Medicine*, 7(11), e984. https://doi.org/10.1002/mgg3.984

The above article, published online on 30 September 2019 in Wiley Online Library (wileyonlinelibrary.com), has been retracted by agreement between the journal Editor in Chief, Suzanne Hart, and Wiley Periodicals LLC. Following publication of this article, concerns were raised by a third party regarding consent and ethical approval for the research undertaken and reported in the article. The publisher and representatives of the journal’s editorial board undertook a review of the consent documentation provided, against the research performed and reported in the article. The review uncovered inconsistencies between the consent documentation and the research reported; the documentation was not sufficiently detailed to resolve the concerns raised. As a result, the parties have made the decision to retract the article. In addition, consent documentation did not give approval for data associated with this article to be shared publicly. The associated data for this article has been withdrawn from the publication record following a concern that the data could be used to identify those who have participated in the research. Jun Yao responded on behalf of all the authors, who disagree with the retraction.

